# Effect of n-6/n-3 PUFA ratio on body fat deposition, tissues fatty acid composition and key genes expression of liver lipid metabolism in silver foxes (*Vulpes vulpes fulva*) during the winter fur-growth period

**DOI:** 10.3389/fvets.2022.986388

**Published:** 2022-10-13

**Authors:** Wei Zhong, Guoliang Luo, Jing Luo, Li Guo

**Affiliations:** ^1^Animal Science and Technology College, Jilin Agricultural Science and Technology University, Jilin, China; ^2^State Key Laboratory of Special Economic Animal Molecular Biology, Institute of Special Animal and Plant Sciences of Chinese Academy of Agricultural Sciences, Changchun, China

**Keywords:** n-6/n-3PUFA, silver fox, body fat deposition, tissues fatty acid composition, genes expression of lipid metabolism

## Abstract

**Objective:**

The proportion of n-6/n-3 polyunsaturated fatty acid (PUFA) plays an important role in regulating lipid metabolism. This study aimed to investigate the effects of dietary n-6/n-3 PUFA ratios on body fat deposition, tissues fatty acid composition, and gene expression of liver lipid metabolism of silver foxes during the winter fur growth period.

**Methods:**

Forty-eight age-matched male silver foxes with similar body weights were randomly divided into four dietary groups for 47 days, which were fed n-6/n-3 PUFA ratio with 3, 18, 41, and 136 experimental diets, respectively.

**Results:**

Dietary n-6/n-3 PUFA ratio did not significantly influence fat deposition parameters except for hepatic fat content. The variation trend of the fatty acid composition of liver, intramuscular fat, and subcutaneous fat in silver fox was directly related to dietary fatty acid content (*p* < 0.05). With the dietary n-6/n-3 PUFA ratio increasing, the expression of liver fatty acid synthase (FAS) mRNA and peroxisome proliferator-activated receptor (PPAR) mRNA exhibited the trend of first decreasing and then increasing (*p* < 0.05), whereas L-fatty acid binding protein (L-FABP) mRNA expression showed a gradual increasing trend (*p* < 0.05).

**Conclusion:**

In summary, silver foxes fed an n-6/n-3 PUFA ratio 18:1 diet (supplementing with 9.38% corn oil and 4.62% soybean oil) was more conducive to lipid decomposition, PUFA transport, and utilization of tissues, thereby meeting it for supplying energy and withstanding the cold.

## Introduction

Polyunsaturated fatty acids (PUFAs), especially the n-3 and n-6 series PUFAs, play an important role in the body's lipid metabolism, gene expression regulation, and fatty acid composition of animal products (1, 2). Because n-6 and n-3 series PUFAs cannot be converted into each other and have to be taken in through food, the balancing of the n-6/n-3 PUFA ratio has attracted much attention lately in improving inflammation and decreasing the risk of metabolic diseases (3–6). Dietary fatty acid composition clearly influenced the body fat composition of fur animals (7–9). The previous studies have shown that n-3 and n-6 PUFA in diets regulate the lipid deposition and oxidation in human and animals, thereby affecting the composition of fatty acids in tissues (10–13). Extensive investigations into gene expression as it relates to lipid metabolism have been conducted in swine, chickens, and geese, among other animals, and have mainly focused on lipid deposition and product quality regulation, among other factors (14–17). The silver fox was a precious fur animal and could utilize a higher content fat diet, but its body could keep healthy and have less ability for body fat deposition, which is rather different from other monogastric animals (18). However, how to utilize and metabolize fatty acids in silver fox have not been investigated. Thus, in this study, we aimed to examine the effect of the dietary n-6/n-3 PUFA ratio on the tissues fatty acid composition, body fat deposition, and expression of key genes of liver lipid metabolism of the silver fox during the winter fur growth period to provide basic data to understand the silver fox's lipid metabolism mechanism.

## Materials and methods

### Ethics approval and consent to participate

All animals used in the study were treated following the guidelines established by the Council of China Animal Welfare. Protocols of the experiments were approved by the Animal Ethics Committee of the Chinese Academy of Agricultural Sciences (CAAS).

### Animals, experimental design, and diets

The experiment was performed at the fur animal breeding base. Forty-eight 157-day-old healthy male silver foxes with an average weight of 5,450 ± 140 g at the fur growth stage were randomly divided into four groups (12 replicates per group, one silver fox per replicate). Each group was provided diets of different lipid compositions, and the feeds had the same ingredients except for the composition and ratio of the lipids. The diet of Group I was supplemented with 12% fish oil and 2% soybean oil, yielding an n-6/n-3 of 3.00; the diet of Group II was supplemented with 9.38% corn oil and 4.62% soybean oil, yielding an n-6/n-3 of 18.03; the diet of Group III was supplemented with 12% corn oil and 2% soybean oil, yielding an n-6/n-3 of 40.83; and the diet of Group IV was supplemented with 1.5% fish oil and 12.5% corn oil, yielding an n-6/n-3 of 136.36.

Each of the experimental animals was raised separately in a cage. The experiment was initiated on October 13th, and completed on December 1st. The pre-feeding period was 7 days, and the formal experimental period was 40 days, during which the animals were fed twice a day (at 8:00 am and 15:00 pm), with free access to water. The ingredients, nutrient levels, and fatty acid compositions of the feeds of the different groups are shown in Tables 1, 2.

**Table 1:** Feed ingredients and chemical composition of experimental diets (air-dry basis) (%).

**Items**	**Groups (n-6/n-3PUFA ratio)**			
	**I (3:1)**	**II (18:1)**	**III (41:1)**	**IV (136:1)**
**Ingredients**				
Extruded corn	32.75	32.75	32.75	32.75
Soybean meal	12.00	12.00	12.00	12.00
Corn protein meal	8.00	8.00	8.00	8.00
DDGS	1.55	1.55	1.55	1.55
Fish meal	16.00	16.00	16.00	16.00
Meat meal	10.00	10.00	10.00	10.00
Lysine	0.80	0.80	0.80	0.80
Methionine	0.40	0.40	0.40	0.40
Premix^1)^	1.00	1.00	1.00	1.00
Fish oil	12.00	0.00	0.00	1.50
Corn oil	0.00	9.38	12.00	12.5
Soybean oil	2.00	4.62	2.00	0.00
CaHPO_4_	3.00	3.00	3.00	3.00
NaCl	0.50	0.50	0.50	0.50
Total	100.00	100.00	100.00	100.00
Chemical composition Gross energy, MJ/kg	19.04	19.03	19.14	19.05
Crude protein	29.76	29.39	29.82	29.76
Ether extract	15.50	15.13	15.77	15.09
Crude carbohydrate	41.43	42.66	41.36	42.44
Lysine^3)^	2.36	2.36	2.36	2.36
Methionine^3^)+Cystine	1.43	1.43	1.43	1.43
Calcium	1.61	1.54	1.50	1.40
Phosphorous	1.14	1.03	1.10	0.98

1) One kilogram of premix contained the following composition: VA, 300,000 IU; VD_3_,200,000 IU; VE, 4,000 mg; VK_3_, 50 mg; VB_1_, 400 mg; VB_2_, 500 mg; VB_6_, 200 mg; VB_12_, 4.2 mg; folic acid, 50 mg; pantothenic acid, 2,200 mg; biotin, 1,600 mg; choline chloride, 120 mg; VC, 12,000 mg; Fe, 4,000 mg; Zn, 3,200 mg; Mn, 1,600 mg; I, 80 mg; Se, 12 mg; and Cu, 500 mg.

2) GE, CP, EE, Lys, Met, Ca and P were measured values, whereas CC was calculated value.

3) Lysine and methionine are feed-grade; content 99%.

**Table 2:** Fatty acid compositions of the experimental diets (%).

**Fatty acids**	**Groups (n-6/n-3PUFA ratio)**			
	**I (3:1)**	**II (18:1)**	**III (41:1)**	**IV (136:1)**
C12:0	0.00	0.00	0.00	0.00
C14:0	2.25	0.01	0.00	0.18
C14:1	0.00	0.00	0.00	0.00
C15:0	0.00	0.00	0.00	0.00
C15:1	0.00	0.00	0.00	0.00
C16:0	13.17	3.90	3.23	3.51
C16:1	6.39	0.01	0.00	0.52
C17:0	0.00	0.00	0.00	0.00
C17:1	0.00	0.00	0.00	0.00
C18:0	2.75	1.04	0.73	0.64
C18:1n9t	0.00	0.00	0.00	0.00
C18:1n9c	47.41	21.63	20.87	22.45
C18:2n6t	0.00	0.00	0.00	0.00
C18:2n6c	20.56	69.29	73.13	71.95
C20:0	0.09	0.13	0.13	0.12
C18:3n6	0.00	0.00	0.00	0.00
C20:1	0.26	0.08	0.09	0.10
C18:3n3	2.97	3.84	1.79	0.19
C21:0	0.00	0.00	0.00	0.00
C20:2n6	0.00	0.01	0.00	0.00
C22:0	0.03	0.05	0.02	0.00
C22:1n9	0.00	0.00	0.00	0.00
C20:3n3	0.00	0.00	0.00	0.00
C23:0	0.00	0.00	0.00	0.00
C20:4n6	0.14	0.00	0.00	0.01
C22:2n6	0.00	0.00	0.00	0.00
C24:0	0.02	0.01	0.01	0.00
C20:5n3	2.96	0.00	0.00	0.24
C24:1	0.03	0.00	0.00	0.00
C22:6n3	0.97	0.00	0.00	0.08
SFA	18.31	5.14	4.12	4.45
MUFA	54.09	21.72	20.96	23.07
PUFA	27.60	73.14	74.92	72.48
n-6	20.69	69.30	73.13	71.96
n-3	6.90	3.84	1.79	0.529
n-6/n-3	3	18	41	136

SFA saturated fatty acid(s), MUFA monounsaturated fatty acid(s), PUFA polyunsaturated fatty acid(s).

### Sample collection

At the end of the experimental period, eight silver foxes from every group were randomly selected and were euthanized. The liver and subcutaneous fats of silver foxes were weighed. 2 g liver sample was taken, rinsed blood stains with normal saline, put into the frozen storage tube, put into liquid nitrogen for more than 10 min, and then moved into −80°C refrigerator storage for gene detection. Samples collected from partly liver were dried to constant weight at 65 degrees and fat content was determined by soxhlet extraction. The animals were sampled for liver, medial thigh muscle, and subcutaneous belly fat 50 g, respectively, which were cleaned by normal saline and stored frozen (−20°C) for fatty acid analysis.

### Chemical analyses

Samples of feed were analyzed for DM, CP, EE, Ca, P, and AA according to AOAC (19) methods. Amino acids were determined by hydrolyzing samples with 6 mol/L HCl for 24 h at 110°C (20) and analyzed using an Amino Acid Analyzer (Hitachi L-8800; Hitachi, Ltd., Tokyo, Japan). Methionine and cysteine were determined as methionine sulfone and cysteic acid after cold performic acid oxidation overnight and hydrolyzed. The GE concentration was measured using an adiabatic bomb calorimeter (C2000, Calorimeter; IKA Company; Germany). Fatty acids were pretreated using the methyl esterification method and analyzed with Gas Chromatography–Mass Spectrometry (GC–MS; Agilent 7890A-7000B, USA) referring to the standard method (21).

### Total RNA extraction and cDNA synthesis in liver samples

The liver samples were pulverized in liquid nitrogen and transferred to a 1.5-ml RNAse-free Eppendorf tube. Total RNA was extracted using the RNAiso Reagent Kit (TaKaRa Corporation) according to the manufacturer's instructions. The extracted total RNA was examined for integrity by gel electrophoresis and for purity by determining absorbance (OD value) at 260 and 280 nm. Reverse transcription was performed using the Reverse Transcription Kit (TaKaRa Corporation), and the reverse transcription products were stored at −80°C for later use.

### Gene expression analysis of liver lipid metabolism

The relative amounts of expression of fatty acid synthase (FAS) mRNA, peroxisome proliferator-activated receptor (PPAR) mRNA and fatty acid binding protein (FABP) were determined through real-time quantitative PCR assay (SYBR Green dye method, Trans-Start kit) using β-actin as the reference gene. The primers were synthesized by the Shanghai Biological Engineering Co., Ltd (Table 3). The PCR reaction (20 μl) comprised the following reagents: 10 μl 2 × Trans Start Top Green qPCR SuperMix, 0.4 μl of each of the two primers (10 μmol/L), 0.4 μl Passive Reference Dye (50×), 7.8 μl of RNAse-free dH_2_O and 1 μl of cDNA. The PCR cycling program was as follows: 95°C for 1 min, followed by 40 cycles of 95°C for 5 s and annealing (with specific annealing temperatures) shown in Table 3 for 5 s. The melting curve was used to determine the specificity of amplicons, using the following program: from 65 to 95°C, an assay was conducted at increments of 0.5°C until reaching 95°C, for a total of 61 cycles.

**Table 3:** Primer sequences and parameters for real-time PCR.

**Genes**	**Primer sequence (5′– 3′)**	**Gene bank No**.	**Product size /bp**
β-actin	F: TGCCCATCTATGAGGGGTATG R: CCTTGATGTCACGCACGATT	XM_041749381	153
FAS	F: GATACCTGTGGTTTTGCGTCC R: CAGCGATGCCGATGATGTAT	XM_026014782	183
PPAR	F: AAAGAGCCTAAGGAAACCG R: GCAAATGATAGCAGCCACA	XM_041757116	345
FABP	F:ACAGACTTGATGCCTTTG R:GAAATCGTGCAGAATGG	NM_001287051	185

### Statistical analyses

Experimental data were exported to EXCEL2003 and collated, and statistical analyses were performed using the GLM program of SAS V8 software; multiple comparisons were performed using Duncan's test, and *p* < 0.05 and *p* > 0.05 were used to determine whether findings were significant or insignificant, respectively. The test measurements were presented in the form of means ± standard deviations.

## Results

### Effects of the dietary n-6/n-3 PUFA ratio on body fat deposition traits of silver foxes

The dietary n-6/n-3 PUFA ratio exerted a significant impact on the hepatic fat content of the silver foxes (*p* < 0.05, Table 4), and the hepatic fat content of the silver foxes of Groups I, III, and IV was significantly higher than that of Group II (*p* < 0.05), whereas the hepatic fat content among the silver foxes of Groups I, III, and IV showed no significant differences (*p* > 0.05). The dietary n-6/n-3 PUFA ratio did not show any significant effect on the hepatic somatic index, liver fat percentage, subcutaneous fat weight, and subcutaneous fat percentage (*p* > 0.05), but of which in Group II was relatively lower than that of the other groups, except for hepatic somatic index.

**Table 4:** Effects of dietary n-6 /n-3 PUFA ratio on body fat deposition traits of silver fox during the winter fur-growing period %.

**Items**	**Groups (n-6/n-3PUFA ratio)**				***P*-value**
	**I (3:1)**	**II (18:1)**	**III (41:1)**	**IV (136:1)**	
Hepatic somatic index	2.78 ± 0.36	3.27 ± 0.32	2.93 ± 0.26	2.78 ± 0.48	0.0938
Hepatic fat content	7.73 ± 0.78^a^	5.50 ± 0.96^b^	7.78 ± 1.53^a^	7.36 ± 1.72^a^	0.0481
Liver fat percentage	1.67 ± 0.58	1.56 ± 0.22	2.21 ± 0.42	2.17 ± 0.57	0.0703
Subcutaneous fat weight/g	310.40 ± 54.03	278.17 ± 61.76	324.00 ± 47.35	301.83 ± 63.20	0.576
Subcutaneous fat percentage	4.40 ± 0.91	4.36 ± 0.90	4.70 ± 0.80	4.94 ± 0.84	0.620

In the same row, values with different superscript mean significant difference (P < 0.05).

### Effects of the dietary n-6/n-3 PUFA ratio on the tissues fatty acids composition of silver foxes

The dietary n-6/n-3 PUFA ratio exerted significant effects on liver SFA, MUFA, PUFA, N-3PUFA, and N-6 PUFA (*p* < 0.05, Table 5). Liver SFA of Group III was significantly lower than those of Groups I and II (*p* < 0.05), whereas no difference from that of Group IV (*p* > 0.05). Liver PUFA and N-6 PUFA of Group III were significantly higher than those of Groups I, II, and IV (*p* < 0.05), whereas groups I, I, and IV were not significantly different (*p* > 0.05). Liver N-6 PUFA of Group II was significantly greater than that of Group I (*p* < 0.05). Liver MUFA of Group IV was significantly higher than that of Groups I and III (*p* < 0.05), whereas no significant difference from Group II (*p* > 0.05), and no significant difference was found among Groups I, II, and III (*p* > 0.05).

**Table 5:** Effects of dietary n-6 /n-3 PUFA ratio on fatty acid profiles of liver of silver fox during the winter fur-growing period (proportion of total fatty acid) %.

**Items**	**Groups (n-6/n-3PUFA ratio)**				***P*-value**
	**I (3:1)**	**II (18:1)**	**III (41:1)**	**IV (136:1)**	
C10:0	0.24 ± 0.14	ND	ND	0.42 ± 0.03	0.1876
C12:0	0.38 ± 0.20^b^	1.04 ± 0.34^a^	0.85 ± 0.13^a^	0.71 ± 0.13^ab^	0.0015
C14:0	0.76 ± 0.21	0.81 ± 0.19	1.00 ± 0.48	0.90 ± 0.23	0.5392
C16:0	15.55 ± 2.48	15.58 ± 1.93	13.43 ± 1.95	17.57 ± 3.66	0.0589
C16:1	2.02 ± 0.51^ab^	1.58 ± 0.63^b^	1.56 ± 0.82^b^	2.50 ± 0.55^a^	0.0447
C17:0	0.69 ± 0.10	0.95 ± 0.20	0.92 ± 0.21	0.76 ± 0.16	0.0567
C18:0	43.03 ± 5.37^a^	41.69 ± 2.83^ab^	37.8 ± 4.23^bc^	36.35 ± 3.02^c^	0.0191
C18:1n9c	9.29 ± 2.75	12.13 ± 2.28	10.60 ± 4.80	13.77 ± 3.15	0.1346
C18:2n6c	13.91 ± 2.21^c^	18.34 ± 2.13^b^	21.73 ± 1.62^a^	18.48 ± 2.32^b^	<0.0001
C20:0	0.35 ± 0.09	ND	ND	0.42 ± 0.09	0.3292
C20:3n3	0.49 ± 0.16	ND	ND	0.47 ± 0.24	0.8980
C20:3n6	0.38 ± 0.13	ND	ND	0.36 ± 0.00	0.8124
C20:4n6	5.31 ± 1.78^b^	6.81 ± 1.83^b^	10.01 ± 2.94^a^	5.30 ± 2.16^b^	0.0039
C22:6n3	5.78 ± 2.36^a^	1.76 ± 0.47^b^	3.21 ± 0.64^b^	2.83 ± 1.05^b^	0.0006
SFA	60.14 ± 5.40^a^	59.60 ± 3.95^a^	53.02 ± 4.26^b^	57.18 ± 3.31^ab^	0.0237
MUFA	11.30 ± 3.22^b^	13.71 ± 2.86^ab^	10.28 ± 3.98^b^	16.27 ± 3.55^a^	0.0222
PUFA	27.77 ± 6.81^b^	26.70 ± 4.35^b^	34.71 ± 5.45^a^	28.46 ± 4.41^b^	0.0477
N-6	19.54 ± 3.85^c^	25.15 ± 375^b^	31.88 ± 4.28^a^	22.50 ± 4.01^bc^	<0.0001
N-3	3.08 ± 0.65^ab^	1.86 ± 0.56^bc^	3.30 ± 0.66^a^	2.11 ± 0.82^c^	0.0102

In the same row, values with different superscript mean significant difference (P < 0.05).

The dietary n-6/n-3 PUFA ratio exerted highly significant effects on intramuscular fat SFA, MUFA, PUFA, N-6 PUFA, and N-3 PUFA (*p* < 0.05, Table 6). Intramuscular fat SFA and N-3 PUFA of Group I was significantly higher than those of the other Groups (*p* < 0.05), but no significant different in SFA was found in Group II, III and IV (*p* > 0.05), whereas N-3 PUFA of Group II was highly significant lower than those of Groups III and IV (*p* < 0.05), and no significant difference was found between Groups III and IV (*p* > 0.05). Intramuscular fat MUFA of Groups I and IV was obviously significantly higher than those of Groups II and III (*p* < 0.05), whereas no significant difference was found between Groups I and IV, between Groups II and III (*p* > 0.05). Intramuscular fat PUFA and N-6 PUFA of Group I was highly significantly lower than those of the other groups (*p* < 0.05), and that of Group II was significantly higher than that of Group IV (*p* < 0.05), but no difference was found from Group III (*p* > 0.05). However, intramuscular fat PUFA and N-6 PUFA among Groups III and IV exhibited no significant differences (*p* > 0.05).

**Table 6:** Effects of dietary n-6 /n-3 PUFA ratio on fatty acid profiles of intramuscular fat of silver fox during the winter fur-growing period (proportion of total fatty acid) %.

**Items**	**Groups (n-6/n-3PUFA ratio)**				***P*-value**
	**I (3:1)**	**II (18:1)**	**III (41:1)**	**IV (136:1)**	
C12:0	0.11 ± 0.03	0.07 ± 0.01	0.09 ± 0.03	0.08 ± 0.02	0.3098
C14:0	2.83 ± 0.29^a^	1.59 ± 0.32^b^	1.66 ± 0.26^b^	1.84 ± 0.34^b^	<0.0001
C14:1	0.28 ± 0.04^a^	0.18 ± 0.08^b^	0.18 ± 0.08^b^	0.19 ± 0.05^b^	0.0242
C15:0	0.17 ± 0.03^a^	0.09 ± 0.02^b^	0.10 ± 0.01^b^	0.12 ± 0.01^b^	<0.0001
C16:0	22.90 ± 0.79^a^	17.15 ± 1.76^b^	17.40 ± 1.32^b^	17.31 ± 1.61^b^	<0.0001
C16:1	9.17 ± 1.39^a^	4.72 ± 1.22^b^	5.19 ± 1.84^b^	5.68 ± 1.33^b^	<0.0001
C17:0	0.22 ± 0.03	0.22 ± 0.09	0.19 ± 0.05	0.17 ± 0.02	0.1970
C18:0	4.97 ± 0.42^a^	4.12 ± 0.63^b^	4.21 ± 0.80^b^	4.12 ± 0.48^b^	0.0399
C18:1n9c	37.46 ± 1.71	36.22 ± 2.73	35.78 ± 2.12	38.94 ± 2.33	0.0644
C18:1n9t	ND	0.09 ± 0.03	0.09 ± 0.04	0.11 ± 0.03	0.6245
C18:2n6c	19.51 ± 1.15^c^	34.31 ± 2.96^a^	33.04 ± 3.33^ab^	29.63 ± 4.59^b^	<0.0001
C18:3n3	0.73 ± 0.11^b^	0.47 ± 0.06^c^	0.99 ± 0.23^a^	0.60 ± 0.23^bc^	0.0002
C20:0	ND	0.13 ± 0.05	0.11 ± 0.05	0.12 ± 0.04	0.9155
C20:1	0.22 ± 0.03^a^	0.15 ± 0.03^bc^	0.14 ± 0.03^c^	0.18 ± 0.02^b^	0.0008
C20:4n6	0.66 ± 0.10^a^	0.66 ± 0.09^a^	0.53 ± 0.13^ab^	0.48 ± 0.16^b^	0.0349
C20:5n3	0.69 ± 0.14^a^	ND	0.16 ± 0.04^b^	0.16 ± 0.03^b^	<0.0001
C22:6n3	0.44 ± 0.04^a^	0.14 ± 0.05^c^	0.18 ± 0.04^bc^	0.22 ± 0.04^b^	<0.0001
SFA	31.10 ± 1.06^a^	23.15 ± 2.62^b^	23.76 ± 1.52^b^	23.75 ± 1.69^b^	<0.0001
MUFA	47.22 ± 2.41^a^	41.25 ± 2.93^b^	41.34 ± 3.40^b^	45.04 ± 3.40^a^	0.0031
PUFA	21.67 ± 1.82^c^	35.50 ± 2.98^a^	34.90 ± 3.37^ab^	31.21 ± 4.95^b^	<0.0001
N-6	20.08 ± 1.27^c^	34.96 ± 2.93^a^	33.60 ± 3.34^ab^	30.11 ± 4.60^b^	<0.0001
N-3	1.86 ± 0.24^a^	0.54 ± 0.07^c^	1.30 ± 0.26^b^	1.10 ± 0.39^b^	<0.0001

In the same row, values with different superscript mean significant difference (P < 0.05).

The dietary n-6/n-3 PUFA ratio exerted significant effects on subcutaneous fat SFA, PUFA, N-6 PUFA, N-3 PUFA, and MUFA (*p* < 0.05, Table 7). Subcutaneous fat SFA of Group I was significantly higher than those of the other Groups (*p* < 0.05), but no significant difference was found among Group II, III, and IV (*p* > 0.05). Subcutaneous fat MUFA of Group I was significantly higher than that those of Group II and III (*p* < 0.05), but no significant difference from Group IV (*p* > 0.05). There was no significant difference in subcutaneous fat MUFA among Group II, III, and IV (*p* > 0.05). Subcutaneous fat PUFA and N-6 PUFA of Group I were significantly lower than those of the other Groups (*p* < 0.05), but no significant difference was found among Group II, III, and IV (*p* > 0.05). Subcutaneous fat N-3 PUFA of Group I was highly significant higher than those of the other groups (*p* < 0.05). Subcutaneous fat N-3 PUFA of Group II was highly significant lower than that of Group IV (*p* < 0.05), but no significant difference from Group III (*p* > 0.05), and that of Group III was significantly lower than that of Group IV (*p* < 0.05).

**Table 7:** Effects of dietary n-6 /n-3 PUFA ratio on fatty acid profiles of subcutaneous fat of silver fox during the winter fur-growing period (proportion of total fatty acid) %.

**Items**	**Groups (n-6/n-3PUFA ratio)**				***P*-value**
	**I (3:1)**	**II (18:1)**	**III (41:1)**	**IV (136:1)**	
C12:0	2.01 ± 0.82^a^	0.08 ± 0.02^b^	0.09 ± 0.01^b^	0.08 ± 0.02^b^	<0.0001
C14:0	2.92 ± 0.66^a^	1.29 ± 0.18^b^	1.44 ± 0.34^b^	1.57 ± 0.17^b^	<0.0001
C14:1	0.18 ± 0.01	0.15 ± 0.05	0.15 ± 0.04	0.17 ± 0.03	0.3869
C15:0	0.12 ± 0.01	0.11 ± 0.02	0.11 ± 0.01	0.11 ± 0.01	0.4082
C16:0	18.36 ± 0.83^a^	14.02 ± 0.70^b^	13.87 ± 1.85^b^	15.12 ± 1.64^b^	0.0002
C16:1	6.08 ± 0.33^a^	3.75 ± 0.45^b^	4.14 ± 1.43^b^	4.89 ± 0.99^ab^	0.0039
C17:0	0.17 ± 0.02	0.15 ± 0.007	0.16 ± 0.03	0.16 ± 0.03	0.5212
C18:0	4.49 ± 1.08^a^	2.85 ± 0.35^b^	3.42 ± 0.65^b^	2.99 ± 0.51^b^	0.0068
C18:1n9c	37.09 ± 1.09	35.25 ± 2.78	34.25 ± 2.13	36.55 ± 1.10	0.1313
C18:2n6c	26.16 ± 2.01^b^	40.91 ± 2.36^a^	40.06 ± 4.62^a^	36.82 ± 3.10^a^	<0.0001
C20:0	0.16 ± 0.04	0.23 ± 0.05	0.20 ± 0.07	0.16 ± 0.02	0.0728
C20:1	0.95 ± 0.07	0.85 ± 0.05	1.69 ± 0.05	0.90 ± 0.07	<0.0001
C20:2n6	0.17 ± 0.02^a^	0.14 ± 0.03^ab^	0.12 ± 0.03^b^	0.13 ± 0.03^b^	0.0329
C20:4n6	0.13 ± 0.02	0.15 ± 0.04	0.12 ± 0.01	0.12 ± 0.05	0.6629
C20:5n3	0.24 ± 0.05	ND	ND	0.15 ± 0.06	0.1113
C22:6n3	0.23 ± 0.04^a^	0.11 ± 0.04^b^	0.12 ± 0.03^b^	0.19 ± 0.06^a^	0.0034
SFA	28.41 ± 3.00^a^	18.72 ± 0.77^b^	19.28 ± 1.77^b^	20.91 ± 1.98^b^	<0.0001
MUFA	44.29 ± 1.42^a^	39.99 ± 2.56^b^	40.04 ± 3.18^b^	42.51 ± 1.26^ab^	0.0164
PUFA	26.93 ± 2.06^b^	41.28 ± 2.39^a^	40.46 ± 4.59^a^	37.31 ± 3.04^a^	<0.0001
N-6	26.47 ± 2.03^b^	41.20 ± 2.39^a^	40.30 ± 4.65^a^	37.05 ± 308^a^	<0.0001
N-3	0.47 ± 0.09^a^	0.11 ± 0.04^c^	0.17 ± 0.11^c^	0.31 ± 0.09^b^	<0.0001

In the same row, values with different superscript mean significant difference (P < 0.05).

### Effects of the dietary n-6/n-3 PUFA ratio on the genes expression of key enzymes of lipid metabolism in the livers of silver foxes

**Figure 1:**
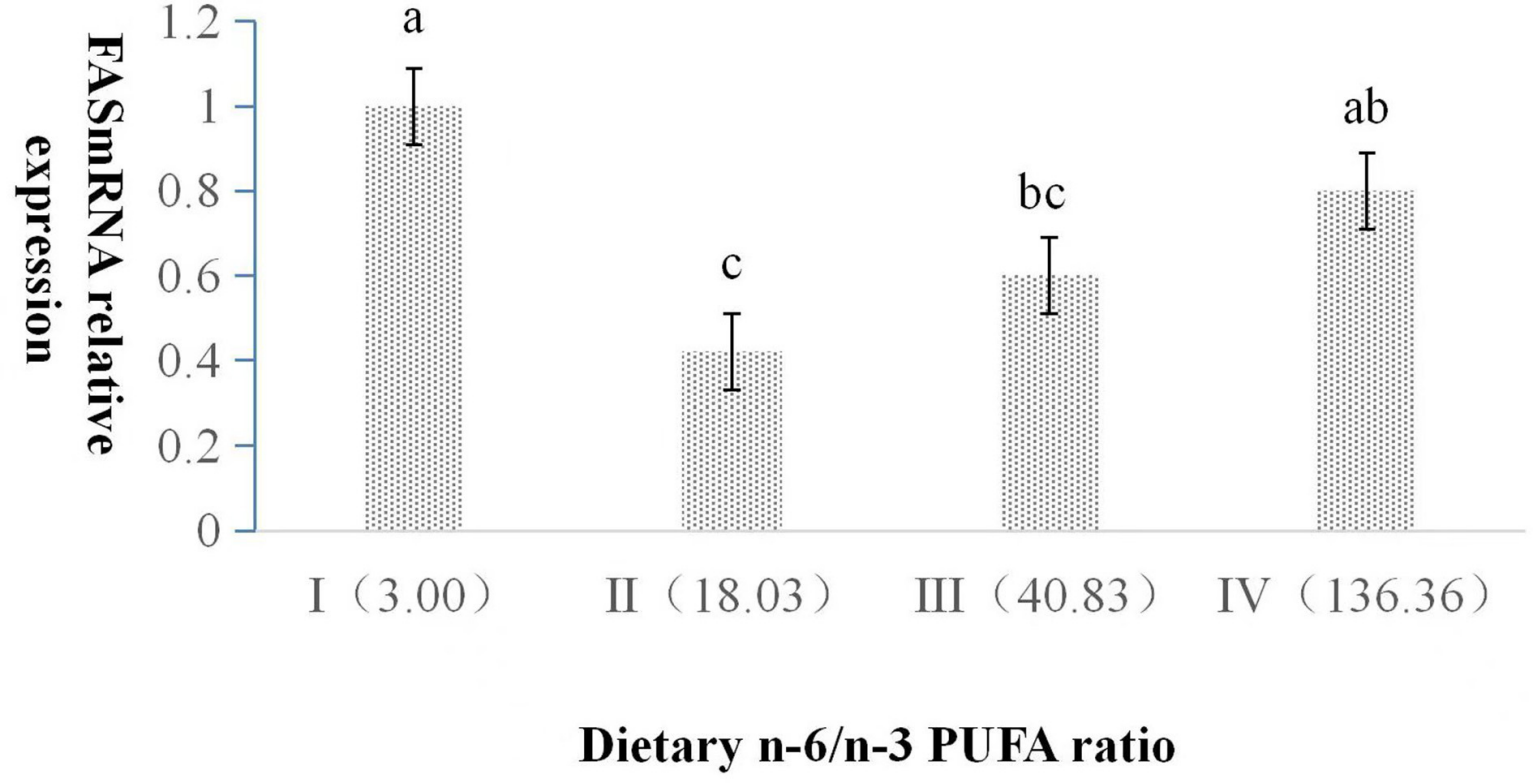
Effects of dietary n-6 /n-3 PUFA ratio on liver FAS mRNA relative expression in silver fox during the winter fur-growth period. Data are presented as the mean ± SD. a,b,c means values with different letters are significantly different (p < 0.05).

The dietary n-6/n-3 PUFA ratio exerted a significant impact on the relative expression level of FAS mRNA (*p* < 0.05, Figure 1). FAS mRNA expression in Group I was significantly higher than those of Group II and III (*p* < 0.05), and no significant difference was found between Group I and IV (*p* > 0.05). FAS mRNA expression in Group II was significantly lower than that of Group IV (*p* < 0.05), and no significant difference was found between Group II and III (*p* > 0.05). There was no significant difference between Group III and IV (*p* > 0.05).

**Figure 2:**
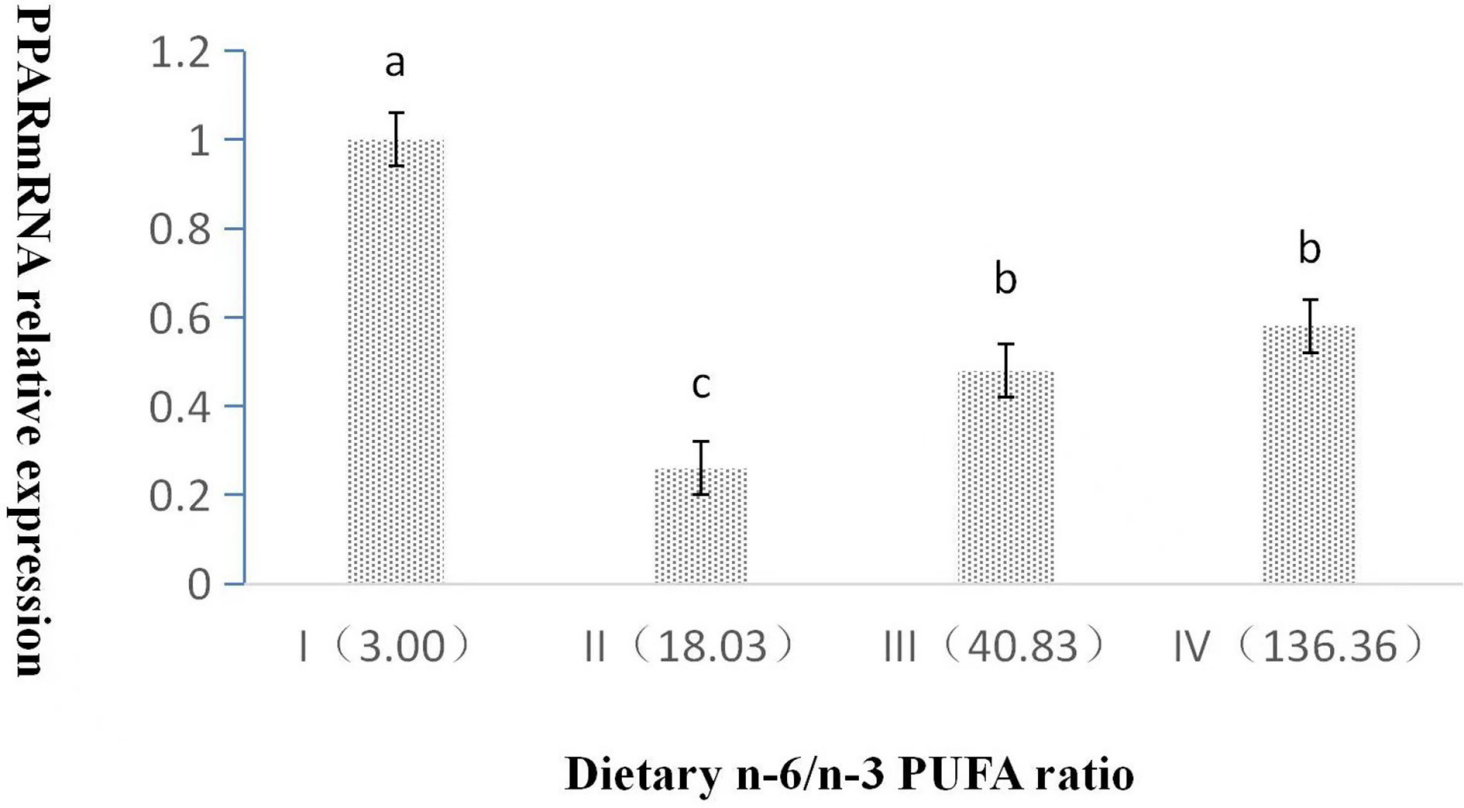
Effects of dietary n-6 /n-3 PUFA ratio on liver PPARmRNA relative expression in silver fox during the winter fur-growth period. Data are presented as the mean ± SD. a,b,c means values with different letters are significantly different (p < 0.05).

The dietary n-6/n-3 PUFA ratio exerted a significant effect on the relative expression level of PPAR mRNA (*p* < 0.05, Figure 2). With an increasing dietary n-6/n-3 PUFA ratio, the relative expression level of PPAR mRNA showed a trend of first declining and then rising, and the relative expression level of PPAR mRNA of Group I was highly significant higher than those of Groups II, III, and IV (*p* < 0.05). Furthermore, the relative expression level of PPAR mRNA of Group III and IV was obviously significantly higher than that of Group II (*p* < 0.05), whereas that of Group III was no significant difference from that of Group IV (*p* > 0.05).

**Figure 3:**
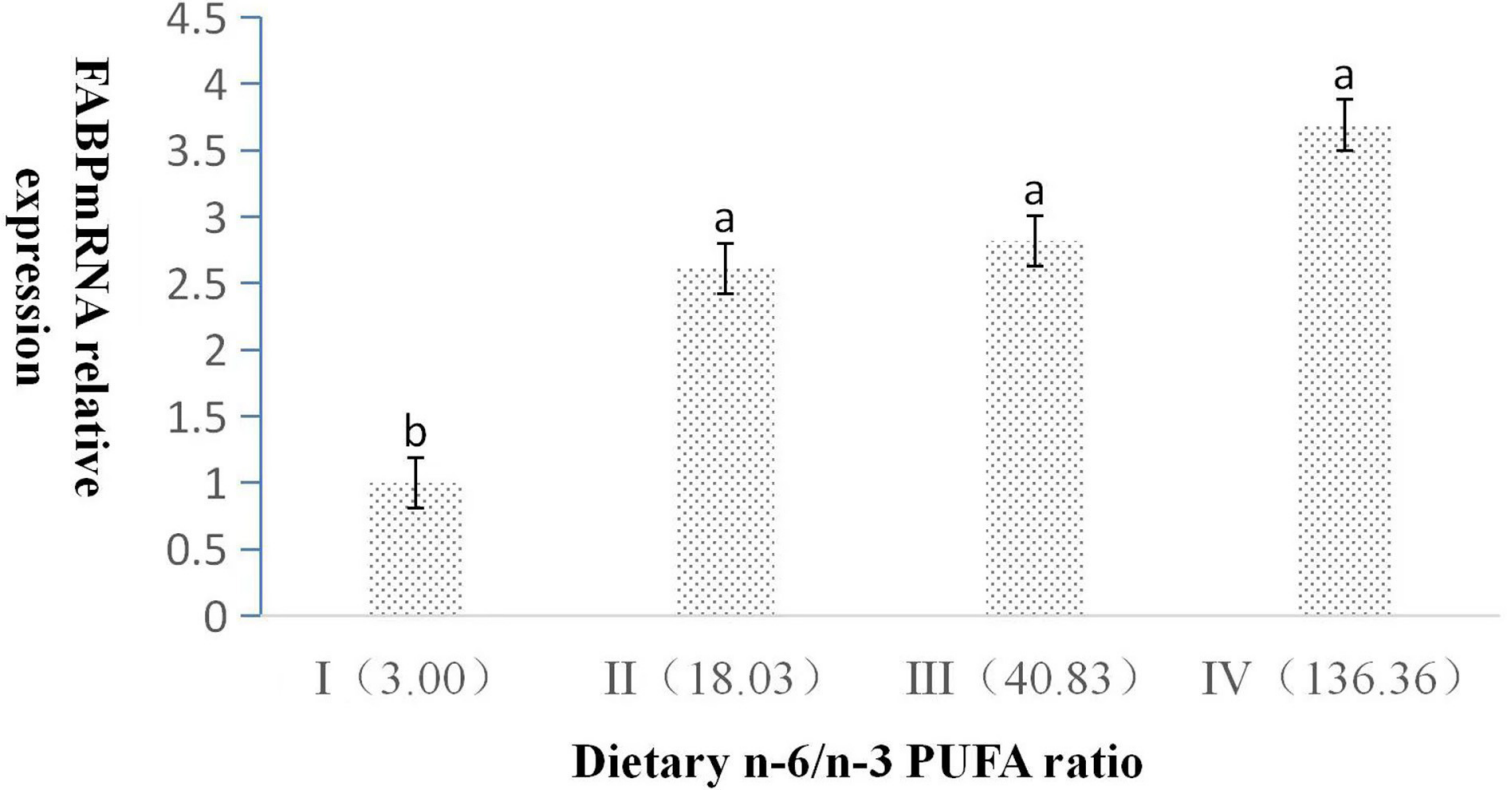
Effects of dietary n-6 /n-3 PUFA ratio on liver FABP mRNA relative expression in silver fox during the winter fur-growth period. Data are presented as the mean ± SD. a,b,c means values with different letters are significantly different (p < 0.05).

The dietary n-6/n-3 PUFA ratio had obviously significant effect on the relative expression level of L-FABP mRNA (*p* < 0.05, Figure 3). With the increasing dietary n-6/n-3 PUFA ratio, the relative expression level of L-FABP mRNA showed a gradual increasing trend. The relative expression level of L-FABP mRNA of Group I was highly significantly lower than those of Group II, III, and IV (*p* < 0.05). L-FABP mRNA expression did not have a significant difference (*p* > 0.05) among Group II, III, and IV.

## Discussion

### Effects of the dietary n-6/n-3 PUFA ratio on the body fat deposition

When the n-6/n-3 PUFA ratio was 18 or 41, the hepatic somatic index of the arctic fox was lower than that of the other n-6/n-3 PUFA ratio (3 or 136); however, the other body deposition indexes were not influenced by the different ratios of n-6/n-3 PUFAs (22). In the present study, hepatic fat content of silver fox fed the diet containing an n-6/n-3 PUFA ratio of 18 was significantly lower than that of the other n-6/n-3 PUFA ratio, whereas body fat deposition indexes were not significantly affected by the dietary n-6/n-3 PUFA ratio in silver fox. This indicated that canine with different genera had similar body fat composition and variation fed the same diet composition. Fish research literatures (23, 24) reported that body fat deposition parameters were not affected by the ratio of n-6/n-3 PUFA, the results of this experiment were consistent with these literatures. The reason that lower hepatic fat content of silver fox fed n-6/n-3 PUFA ratio of 18 might increase the transport of long-chain fatty acids in the liver and reduce the formation of cholesterol and triglycerides in the liver by the expression of lower FAS mRNA and higher L-FABP in liver in the present study.

### Effects of the dietary n-6/n-3 PUFA ratio on the tissues fatty acids composition and contents

The dietary fatty acid composition clearly influences the body fat composition of fur animals (7–9). There are differences in the composition of fatty acids in different tissues of fur animals (10, 25). Body tissues' fatty acid composition in pig and fish was greatly influenced by the feed fatty acid composition and to some extent can reflect the fatty acid composition in feed (3, 26, 27). In this study, we found that the variation trends of the fatty acid composition of liver, intramuscular fat, and subcutaneous fat in silver fox were directly related to dietary fatty acid content, which was consistent with the previous literatures. The content change of SFA, MUFA, and PUFA in liver, intramuscular, and subcutaneous fat of silver fox was consistent with the arctic fox (22, 28). From subcutaneous fat, intramuscular fat to liver in silver fox, SFA content showed a gradual increase trend, whereas UFA (MUFA plus PUFA) content showed a gradual decrease trend, which was consistent with the result (8). These indicated that as compared to SFA, the UFA under its intramuscular and subcutaneous fat was more conducive to oxidating and decomposing to supply the silver fox's energy need. The selective deposition of fat in body tissues was an inherited morphological characteristic in adaptive evolution (29).

### Effects of the dietary n-6/n-3 PUFA ratio on the expression of lipid metabolism-related genes in the livers

An elevated FAS expression level significantly increases the deposition of triglycerides in the body, leading to obesity (30). PUFAs significantly inhibited the activity of fatty acid synthesis in rat liver, and also proved that n-3 PUFAs are more effective than n-6 PUFAs in inhibiting the transcription of FAS gene (31, 32). The previous studies show with the dietary n-6/n-3 PUFA ratio increasing, the expression of FAS genes related to liver fat synthesis decreased first and then increased in *Lateolabrax maculatu* (27). In this study, we found that the hepatic level of FAS mRNA expression exhibited the trend of first decreasing and then increasing with the n-6/n-3 PUFA increasing, and the relative expression level of FAS mRNA was the highest in Group I for silver foxes, which was consistent with the previous literatures. This may be due to lower PUFA content in Group I diet compared to the other three Groups. From Group II to IV, n-3 PUFA content gradually decreased, but PUFA content was similar, which might result in the change of FAS mRNA expression. When dietary n-6/n-3 PUFA was 18, FAS mRNA expression was the lowest, which is consistent with the result of the liver fat content, indicating that proper n-6/n-3 PUFA ratio was beneficial to lipid metabolism, thereby keeping the healthy stage of silver fox.

Liver PPAR regulates the transport of fatty acids to mitochondria by inducing the expression of liver-specific carnitine palmitoyltransferase to stimulate the β-oxidation process and reduce the synthesis of fatty acids and triglycerides (33). PUFA inhibited the expression of related genes in the process of fat synthesis and promotes the process of fat oxidation by acting on liver PPAR (34). The lowering of the dietary n-6:n-3 PUFA ratio might stimulate PPAR target gene expressions (35–37). In this study, the results showed that liver PPAR mRNA expression exhibited the trend of first decreasing and then increasing with increasing n-6/n-3 PUFA ratio. PPAR mRNA expression in Group I was the highest probably due to lower n-6 PUFA and higher n-3PUFA contents, which was consistent with the literature. PPAR mRNA expression change was basically the same with the dietary PUFA content from Group II to IV, which was consistent with the previous literatures, and confirmed that PUFAs could effectively activate PPARs (38).

The previous studies found that knocking out the L-FABP gene can induce liver cholesterol and triglyceride accumulation (39) and L-FABP has a high affinity for long-chain (>C14) fatty acids, which had an important role in absorbing and transferring fatty acids (40–42). In this study, L-FABP mRNA expression showed a gradual increasing trend from Group I to IV with the n-6/n-3 PUFA ratio increasing, which indicated higher PUFA content increasing the expression of L-FABP (28, 43).

## Conclusion

In summary, silver foxes fed an n-6/n-3 PUFA ratio 18:1 diet (supplementing with 9.38% corn oil and 4.62% soybean oil) was more conducive to improving the expression of lipolysis genes, facilitating the lipid decomposition, transporting, and utilizing fatty acids, thereby to meeting the physiological needs of silver foxes for supplying energy and withstanding the cold during the winter fur-growth period.

## Data availability statement

The original contributions presented in the study are included in the article/supplementary material, further inquiries can be directed to the corresponding author/s.

## Ethics statement

The animal study was reviewed and approved by the Animal Ethics Committee of the Chinese Academy of Agricultural Sciences (CAAS). Written informed consent was obtained from the owners for the participation of their animals in this study.

## Author contributions

WZ conceived, designed, and wrote the paper. JL provided technical guidance for data detection. GL and LG participated in the design of the study and contributed to the acquisition of data. All authors reviewed and approved the final manuscript.

## Funding

This work was supported by the Start-up Fund for Scientific Research of Jilin Agricultural Science and Technology University (2021–7008) and the Jilin Province Science and Technology Department foundation Project (20140101033JC).

## Conflict of interest

The authors declare that the research was conducted in the absence of any commercial or financial relationships that could be construed as a potential conflict of interest.

## Publisher's note

All claims expressed in this article are solely those of the authors and do not necessarily represent those of their affiliated organizations, or those of the publisher, the editors and the reviewers. Any product that may be evaluated in this article, or claim that may be made by its manufacturer, is not guaranteed or endorsed by the publisher.
